# Influence of *In Vitro* Digestion on Dipeptidyl Peptidase-IV (DPP-IV) Inhibitory Activity of Plant-Protein Hydrolysates Obtained from Agro-Industrial By-Products

**DOI:** 10.3390/foods13172691

**Published:** 2024-08-26

**Authors:** Raúl Pérez-Gálvez, Carmen Berraquero-García, J. Lizeth Ospina-Quiroga, F. Javier Espejo-Carpio, M. Carmen Almécija, Antonio Guadix, Pedro J. García-Moreno, Emilia M. Guadix

**Affiliations:** Department of Chemical Engineering, University of Granada, 18071 Granada, Spain; rperezga@ugr.es (R.P.-G.); carbegar@ugr.es (C.B.-G.); e.lospinaq@go.ugr.es (J.L.O.-Q.); fjespejo@ugr.es (F.J.E.-C.); mcalmeci@ugr.es (M.C.A.); aguadix@ugr.es (A.G.); eguadix@ugr.es (E.M.G.)

**Keywords:** plant by-products, protein hydrolysates, diabetes mellitus, bioactive peptides, DPP-IV inhibition, INFOGEST, simulated digestion

## Abstract

This study investigates the production of protein hydrolysates with dipeptidyl peptidase-IV (DPP-IV) inhibitory activity from agro-industrial by-products, namely olive seed, sunflower seed, rapeseed, and lupin meals, as well as from two plant protein isolates such as pea and potato. Furthermore, the effect of simulated gastrointestinal digestion on the DPP-IV inhibitory activity of all the hydrolysates was evaluated. Overall, the lowest values of IC_50_ (1.02 ± 0.09–1.24 ± 0.19 mg protein/mL) were observed for the hydrolysates with a high proportion of short-chain [<1 kDa] peptides (i.e., olive seed, sunflower seed, and lupin) or high content of proline (i.e., rapeseed). Contrarily, the IC_50_ of the pea and potato hydrolysates was significantly higher (1.50 ± 0.13–1.93 ± 0.13 mg protein/mL). In vitro digestion led to an increase in peptides < 1 kDa for almost all hydrolysates (except olive and sunflower seed meals), which was noticeable for rapeseed, pea, and potato hydrolysates. Digestion did not significantly modify the DPP-IV inhibitory activity of olive, sunflower, rapeseed, and potato hydrolysates, whereas a significant decrease in IC_50_ value was obtained for pea hydrolysate and a significant increase in IC_50_ was obtained for lupin hydrolysate. Thus, this work shows the potential of agro-industrial by-products for the production of protein hydrolysates exhibiting DPP-IV inhibition.

## 1. Introduction

*Diabetes mellitus* (DM) is a widespread metabolic syndrome characterized by high blood sugar levels, which has reached a global epidemic dimension over the past years. Its side effects, including, for example, limb loss, vision impairment, kidney dysfunction, and heart disease, not only have an impact on the daily life of patients but may pose a severe threat [1]. According to the International Diabetes Federation (IDF), the prevalence of diabetes had risen to 537 million by 2021. The combined impact of diabetes and diabetes-related kidney disease was responsible for around 6.7 million people’s deaths in 2021 [2]. DM is categorized as type 1 diabetes mellitus (T1DM) and type 2 diabetes mellitus (T2DM), the latter affecting 95% of the patients. T1DM is caused by an attack of the immune system on pancreatic β-cells, leading to insulin deficiency. T2DM, a complex metabolic disorder, is characterized by insulin resistance in the liver and muscles, along with excessive glucose production and pancreatic β-cell dysfunction [1]. Moreover, T2DM can also lead to obesity, hypertension, and dyslipidemia (i.e., high levels of triglycerides and low levels of high-density lipoproteins (HDL)) [3]. There is growing concern about the high prevalence of T2DM, which continues to escalate due to the surge in obesity, physical inactivity, and inadequate nutritional habits. In this regard, 700.2 million people are expected to suffer from DM worldwide by 2024, which represents an increase of 30% with respect to 2021 (537 million people) [3]. Lack of physical activity, obesity, and inadequate dietary habits trigger excessive production of reactive oxygen species (ROS), resulting in persistent oxidative stress. This disrupts insulin secretion from the pancreas and hormonal activity in target cells, increasing the likelihood of macro- and micro-vascular complications. Studies indicate that pancreatic β-cells have limited levels of antioxidant enzymes, making them particularly vulnerable to oxidative stress [4].

In light of the above, efforts are ongoing to develop both preventive strategies and therapeutic approaches to tackle diabetes and its associated complications [1]. A number of pharmacologic therapies are currently available for T2DM patients based on (i) direct insulin administration, (ii) promoting insulin secretion, (iii) improving insulin sensitivity, and (iv) modulating metabolism and intestinal absorption of carbohydrates [5]. Among the glucose-lowering approaches, some drugs act as inhibitors of α-glucosidase and α-amylase in the digestive tract, reducing glucose uptake. Regular consumption of synthetic α-glucosidase and α-amylase inhibitors like acarbose, miglitol, and voglibose may lead to undesired gastrointestinal effects. Another common oral therapy for T2DM is metformin, which is effective in regulating glucose production in the liver. However, this medication is contraindicated in patients with moderate to severe chronic kidney failure. In this line, sulfonylureas have also been employed to treat T2DM since they stimulate insulin secretion, but their administration is linked to side effects such as hypoglycemia or weight gain. In this sense, the current research on T2DM treatment has led to emerging drug therapies like glucagon-like peptide 1 (GLP-1) agonists and dipeptidyl peptidase IV (DPP-IV) enzyme inhibitors [3,6]. Concerning the latter, common DPP-IV inhibitors (e.g., alogliptin, linagliptin, saxagliptin, sitagliptin, and vildagliptin) enhance the levels of incretin hormones, promoting glucose homeostasis and appetite suppression. Nevertheless, like other synthetic drugs for treating T2DM, they present some disadvantages. For instance, patients may experience changes in weight, stomach discomfort, low blood sugar levels (hypoglycemia), and even renal or hepatic damage [6].

As an alternative to synthetic drugs, food-derived natural peptides have gained significant interest for their distinctive ability to regulate blood glucose levels (e.g., by inhibiting the DPP-IV enzyme) [7,8]. In this sense, antidiabetic peptides derived from food sources present few side effects compared to commercial medications. So far, they have been successfully isolated through enzymatic hydrolysis from both animal or vegetal protein sources, like milk, meat products, plants, and marine organisms. All these proteins are reported as sources of bioactive peptides [8]. More specifically, plant-derived proteins are widely reported as a sustainable source for the production of antidiabetic peptides. To mention a few works, Thakur et al. [9] evaluated the inhibitory potential of peptides from *Picrorhiza kurroa* against the DPP-IV enzyme (DPP-IV IC_50_ value of 6.25 µg/mL). Similarly, Majid et al. [10] studied the DPP-IV inhibitory properties of peptides obtained from *Sorghum bicolor* hydrolysates and their in vitro digests, reporting an IC_50_ value of 8.43 µg/mL for the sequence QLRDIVDK.

In any case, it should be noted that, to exert their biological activity, peptides need to resist the attack of gastrointestinal proteases during digestion [11]. In this regard, in vitro assays simulating gastrointestinal stages are useful to evaluate the resistance of DPP-IV inhibitory hydrolysates or peptides against digestion prior to reaching the bloodstream [12,13]. To our knowledge, only a few studies conducted on DPP-IV inhibitory peptides investigate their bioactivity throughout the gastrointestinal process. For instance, Wang et al. [14] investigated the DPP-IV inhibitory activity of hydrolysates from oat, buckwheat, and highland barley meals, as well as their activities after in vitro gastrointestinal digestion of the cereal meals. The latter exhibits an IC_50_ value ranging from 1.98 to 3.91 mg/mL (buckwheat, oat, and highland barley, respectively). Similarly, Dugardin et al. [15] conducted an explorative screening of bioactivities generated by plant-based proteins (i.e., pea, wheat, potato, fava bean, and oat) before and after in vitro static gastrointestinal digestion. Regarding DPP-IV activity, simulated gastrointestinal digestion led to improved DPP-IV inhibition (IC_50_ between 0.54 and 2.03 mg/mL) for all the plant proteins studied. The authors concluded that some of the digested proteins exhibited a remarkable potency as DPP-IV inhibitors, comparable to the results reported from animal sources such as whey protein.

This work aimed at investigating the production of protein hydrolysates exhibiting DPP-IV inhibitory activity from sustainable plant substrates. Moreover, we evaluated the effect of gastrointestinal digestion on the DPP-IV inhibitory activity of the hydrolysates. To this end, a set of hydrolysates was obtained from sunflower seed, olive seed, rapeseed, and lupin meals. These substrates are common agro-industrial by-products in the Mediterranean regions. For the sake of comparison, two plant protein isolates from pea and potato were also considered in this study. Both isolates contained over 70%wt of protein, with little presence of fibers and other non-protein impurities, which were not removed from the rest of plant meals. Thus, this work contributes to our understanding of the production of plant protein hydrolysates inhibiting DPP-IV activity, as well as the influence of digestion on their activity to control the glycemic index.

## 2. Materials and Methods

### 2.1. Materials

Lupin (*Lupinus albus*) was obtained from Dayelet (Barcelona, Spain) and olive seed (*Olea europaea*) meal from Q’omer (Valencia, Spain). Sunflower seed (*Helianthus annuus*) and rapeseed (*Brassica napus*) meals were purchased from Bernabé Campal (Salamanca, Spain). The protein content of these plant substrates was evaluated by the Dumas combustion method [16], as described in Section 2.4.1. The average protein content was 28.4%wt for lupin, 20.9%wt for olive seed, 24.6%wt for sunflower, and 34.5%wt for rapeseed. Protein isolates from pea (*Pisum sativum*) and potato (*Solanum tuberosum*) were provided by Trades (Barcelona, Spain). These substrates displayed average protein contents of 70.9 and 73.6%wt, respectively. The meals and protein isolates were used as received without any further separation of the protein fraction. The plant protein hydrolysates were produced utilizing two commercial endoproteases: Alcalase 2.4 L (subtilisin EC 3.4.21.62) and PTN 6.0S (trypsin EC 3.4.21.4), both obtained from Novozymes (Bagsvaerd, Denmark). All chemicals employed during analysis were of analytical grade, purchased from Sigma-Aldrich (Merk, Madrid, España).

### 2.2. Production of Hydrolysates

The enzymatic hydrolysis of the meals and isolates was carried out in a laboratory-scale jacketed glass reactor. The samples were dissolved in distilled water at a protein concentration of 2.5% *w*/*v* and then transferred to a 250 mL jacketed reactor, where the reaction was conducted at 50 °C and constant magnetic stirring. The pH was adjusted to 8.0 using 0.5 M NaOH and maintained throughout the reaction using a pH-stat titrator (718 STAT Titrino, Metrohm, Herisau, Switzerland). An enzyme mixture of Alcalase 2.4 L and PTN at a 1:1 ratio was utilized as the catalyst at an enzyme-to-substrate ratio of 3% (*w*/*w*). Both enzymes are serine endoproteases, where Alcalase 2.4 L (subtilisin) cleaves a broad range of peptide bonds while PTN (trypsin) presents narrow specificity towards Arg or Lys residues. The combination of 1:1 subtilisin/trypsin has been used in previous works of our research group to produce protein hydrolysates with biological activities from either animal (e.g., fish muscle, insect or blood protein) [17,18,19] or plant protein sources [20,21].

The hydrolysis reactions were carried out in duplicate to verify reproducibility. The reactions were allowed to proceed until reaching a degree of hydrolysis of 20%. Finally, the hydrolysis process was terminated by heating the reaction mixture to 90 °C for 10 min to inactivate the enzymes. The samples were then vacuum-filtered and freeze-dried using a LyoMicron freeze-dryer (Coolvacuum Technologies, Barcelona, Spain). The dried powders obtained from the two replicated hydrolysis processes were combined and stored at −20 °C prior to analysis.

The degree of hydrolysis (*DH*) can be linked to the quantity of 0.5 N NaOH used in the reaction to maintain a constant pH during the process, according to the pH-Stat method [22] as shown in Equation (1):

(1) $$DH,\%=\frac{V_{b}\cdot N_{b}}{\alpha \cdot m_{P}\cdot h_{tot}}\cdot 100$$

where the volume and normality of the base (NaOH) used for the titration are expressed as *V_b_* (mL) and *N_b_* (eq/L), respectively. The mass of the protein present in the reaction mixture (g) is denoted as the term *m_p_*, and *h_tot_* represents the number of peptide bonds per mass of protein, assumed to be 8.6 milliequivalents of peptide bonds per gram of protein. The parameter *α* represents the estimated average extent of dissociation of the α-amino groups at the reaction conditions of pH 8.0 and 50 °C, which was determined to be approximately 88.5% [20]. *DH* 20% leads to a peptide distribution with an average peptide chain length (PCL) of 5 residues. Such short-chain peptides are reported to present better accessibility to DPP-IV catalytic sites, which results in a higher inhibitory effect [6]. It is expected that increasing *DH* increases the proportion of short-chain peptides. Nevertheless, we did not consider testing *DH* above 20% for two reasons: (i) increased duration of the hydrolysis reaction, above 8 h in some cases (e.g., lupin), and (ii) extensive hydrolysis above *DH* 20% generates large amounts of free amino acids that cannot bind effectively to DPP-IV active sites [23].

### 2.3. Simulated In Vitro Digestion

The simulated digestion was carried out following the INFOGEST static protocol [24]. The simulated digestion was divided into three stages: oral, gastric, and intestinal. Each stage was carried out in triplicate in a temperature-controlled shaker (Heidolph, Swabach, Germany) at 37 °C with 250 rpm shaking. Briefly, the initial food substrate was prepared by mixing freeze-dried protein hydrolysates with 7.5 mL of distilled water at a concentration of 50 mg/mL. For the oral digestion stage, the food was mixed with the simulated salivary fluid at a ratio of 1:1 *v*/*v* and maintained at pH 7 for 2 min after the addition of salivary amylase (75 U/mL). Subsequently, the oral bolus was combined with the simulated gastric fluid (1:1 *v*/*v*), the pH was adjusted to 3 and then shaken with pepsine from porcine (2000 U/mL) for 2 h. Following completion of gastric digestion, the simulated intestinal fluid was added (1:1 *v*/*v*); pH was adjusted to 7, and pancreatin (100 U/mL) and bile extract from porcine (0.01 mM) were added. Samples underwent further incubation for 2 h. After completing the digestion, the proteolysis was stopped by heating at 85 °C for 15 min to deactivate enzymes [25]. The digests were then centrifuged at 5000× *g* for 20 min and stored at −20 °C.

### 2.4. Characterization of Hydrolysates and Digests

#### 2.4.1. Protein Content

The nitrogen content of the plant protein hydrolysates was determined in triplicate using the Dumas method [16] with a Flash 2000 CHNS/O elemental analyzer (Thermo Scientific, Waltham, MA, USA). Briefly, samples were weighed and then subjected to complete combustion. The resulting analyte gases were separated by gas chromatography (GC), and the electrical signal of the combustion products (CO_2_, H_2_O, N_2,_ and SO_2_) provided by a thermal conductivity detector was related to the elemental concentration (C, H, N, and S) [26]. Protein content was calculated assuming a nitrogen-to-protein factor of 5.3 [27].

#### 2.4.2. Molecular Weight Distribution

The molecular weight distribution of the plant protein hydrolysates and their digests was obtained using size exclusion chromatography (SEC). The freeze-dried hydrolysates were dissolved in distilled water to a final concentration of 10 mg/mL. Subsequently, the samples were centrifuged at 6000 rpm for 10 min, and the supernatant was filtered through single-use filters with a pore size of 0.22 µm. Following this, 500 µL of each solution was injected into an AKTA Purifier 10 FPLC System equipped with a Superdex Peptide 10/300 GL column (GE Healthcare, Uppsala, Sweden). MiliQ water was used as the mobile phase at a flow rate of 0.5 mL/min during elution. The absorbance of the eluted samples was measured at a wavelength of 280 nm, and the molecular weight distribution of the peptides was expressed as the percentage area under the curve.

#### 2.4.3. Amino Acid Composition

The amino acid profile of the plant protein hydrolysates was estimated by ion exchange chromatography using an automatic amino acid analyzer Biochrom 30 with ion exchange chromatography (Biochrom, Cambridge, UK), according to Moore et al. [28]. The samples were hydrolyzed in 6 M HCl at 110 °C for 21 h. Known amounts of Standard (Nleu) were prepared to correct possible amino acid losses and underwent the same treatment as the samples. After hydrolysis, the samples were cooled to room temperature (20 °C), dissolved in sodium citrate loading buffer (pH 2.2) along with the hydrolyzed Standard, and then analyzed for their amino acid profile through ninhydrin color reaction and photometric detection at 570 nm, except for proline, which was detected at 440 nm. The results were expressed as molar percentage.

#### 2.4.4. DPP-IV Inhibitory Activity

The in vitro DPP-IV inhibition assay for the plant protein hydrolysates and their digests was conducted following a modified protocol based on Lacroix and Li-Chan [29]. To this end, a set of aqueous solutions was prepared by dispersing the powdered plant protein hydrolysates in distilled water at different concentrations ranging from 0.01 to 1 mg protein/mL. One hundred microliters of hydrolysate solution were incubated at 37 °C for 10 min with 25 μL of DPP-IV enzyme (0.02 U/mL). After incubation, the reaction was initiated by adding 50 μL of 1 mM Gly-Pro-p-nitroanilide. The extent of the reaction was monitored by measuring the absorbance at 405 nm for 2 h at 37 °C, employing a Multiskan FC microplate photometer (Thermo Scientific, Vantaa, Finland). Each sample was analyzed in triplicate, and the inhibition activity was calculated by comparing the reaction progress to a control (distilled water), as shown in Equation (2):

(2) $$DPP-IV\;inhibition,\;\%=\left(1-\frac{\rho_{i}}{\rho_{0}}\right)\cdot 100$$

where *ρ_i_* is the slope of the absorbance at 405 nm against time for the sample containing the inhibitor (hydrolysate) and *ρ_0_* is the slope obtained for the negative control (i.e., distilled water instead of hydrolysate solution). The half-maximal inhibitory concentration (IC_50_) value for each sample was calculated. Results are expressed in mg of protein/mL as mean ± standard deviation. The tripeptide Ile-Pro-Ile was included as a positive control, yielding an IC_50_ value of 0.005 mg/mL.

### 2.5. Statistical Analysis

Statistical analysis was carried out on Statgraphics Centurion XVI (Statgraphics, The Plains, VA, USA). One-way analysis of variance and Tukey’s multiple comparison tests were conducted to investigate the effects of plant protein source and in vitro digestion on the molecular weight distribution and DPP-IV inhibitory activity of the hydrolysates. Data are presented as the mean ± standard deviation with a 95% confidence level.

## 3. Results and Discussion

### 3.1. Hydrolysis Curves

Plant proteins are widely used as functional ingredients for food formulations. However, their use is hindered by their poor aqueous solubility, heterogenous composition, and susceptibility to processing conditions. Moreover, the in vitro digestibility of plant proteins is often reduced by the presence of antinutritional factors or their low accessibility to digestive proteases within the vegetal matrix. Both the technofunctional properties and the digestibility of plant proteins can be significantly improved by a range of physical, chemical, or biological approaches, such as enzymatic modification [30]. In this regard, the extension and rate of in vitro digestion is significantly increased on protein hydrolysates compared to intact protein [31]. In our case, the plant substrates were hydrolyzed enzymatically in order to release DPP-IV inhibitory peptides. Subsequently, the hydrolysates were subjected to in vitro digestion to evaluate their resistance or not to digestive proteases.

**Figure 1:**
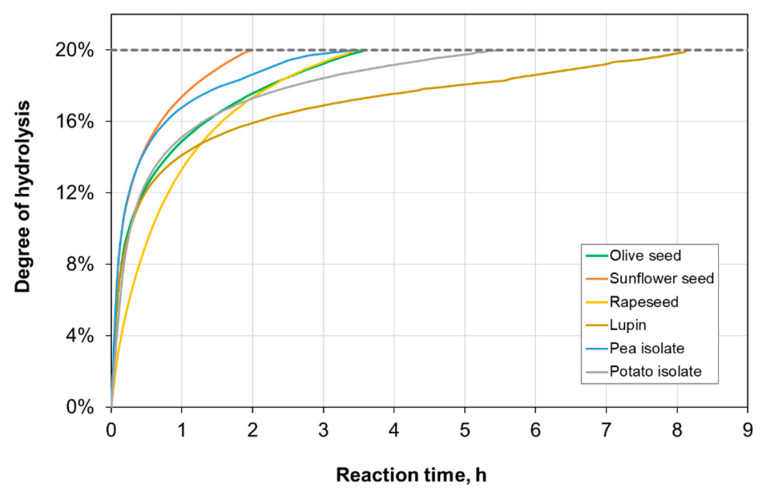
Hydrolysis curves of the plant protein substrates at 50 °C, pH 8, and enzyme (subtilisin-trypsin 1:1)-to-substrate ratio 3%. The degree of hydrolysis was computed as an average of two replicates with standard deviation SD ≤ 0.5%.

Figure 1 shows the hydrolysis curves (i.e., DH against time) of the plant protein substrates. All the hydrolysates were produced employing the same enzyme (subtilisin-trypsin 1:1)-to-protein ratio of 3%. Overall, the hydrolysis curves presented an initial steep increase in DH within the first hour of the reaction, followed by a progressive stabilization of the proteolysis rate. This sigmoidal shape is mainly due to the decrease in peptide bonds available for enzyme attack, although other phenomena, such as product inhibition or loss of enzyme activity, may be present [22]. The plant substrates assayed in this work showed different degradability against protease attack, requiring different reaction times to achieve the target DH 20%. Potato protein isolate and lupin meal proteins were the more resistant against proteolysis, completing the hydrolysis after 5.5 h and more than 8 h, respectively. In the case of potato protein isolate, this can be attributed to the presence of protease inhibitors (e.g., serine-, cysteine-, aspartate- or metalloprotease-inhibitors), which represent 30–40% wt. of the total protein content [32,33]. Lupin seed meal presented 28.4% wt. of protein on a dry basis, mostly composed of globulins (i.e., β-conglutin, α-conglutin, and other minor fractions) [34,35]. The low proteolysis rate observed for this substrate can be attributed to its high content of dietary fiber and other polysaccharides. Such components hamper the accessibility of enzymes to proteins, which are normally entrapped in supramolecular structures [31].

### 3.2. Molecular Weight Distribution and Amino Acid Composition

**Figure 2:**
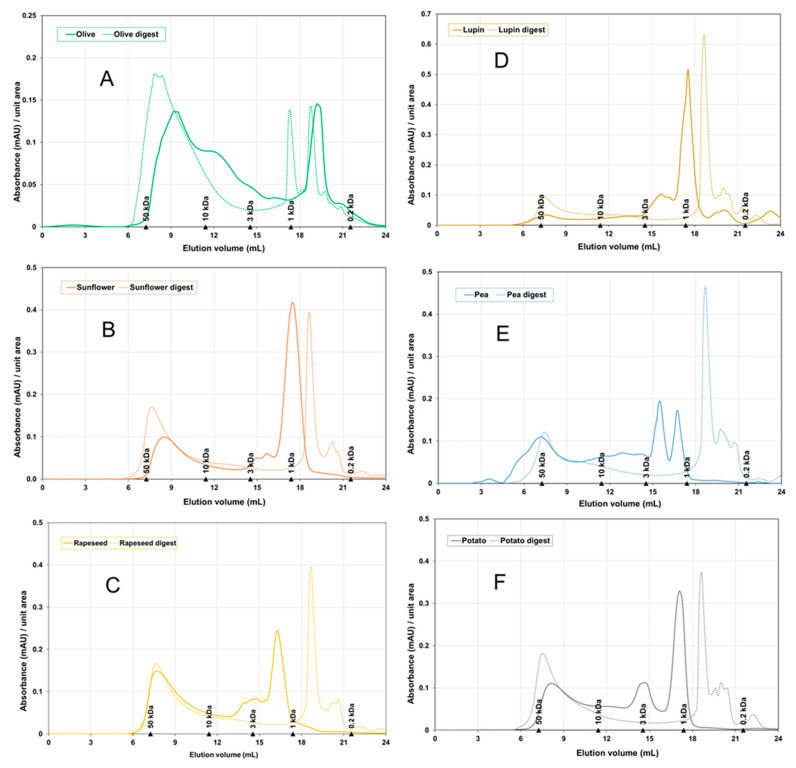
Comparison of the SEC profiles of hydrolysates (solid lines) versus in vitro digests (dotted lines) for (**A**) olive seed meal, (**B**) sunflower seed meal, (**C**) rapeseed meal, (**D**) lupin meal, (**E**) pea protein isolate, (**F**) potato protein isolate. Actual absorbance values (mAU) at 285 nm were normalized so that the total area below the curve is unitary.

**Figure 3:**
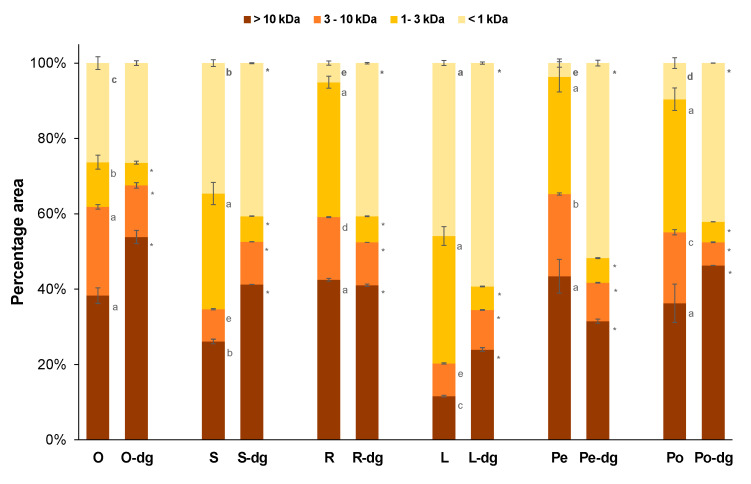
Molecular weight distribution of the plant protein hydrolysates at DH 20% versus their digests. Different superscript letters indicate significant differences (*p* < 0.05) among protein sources for the same MW fraction. (*) indicates significant differences (*p* < 0.05) between the crude hydrolysate and its digest. Legends: O = olive, S = sunflower, R = rapeseed, L = lupin, Pe = pea isolate, Po = potato isolate, -dg = digest.

Figure 2 shows the size exclusion chromatograms (SEC) for the plant protein hydrolysates and their digests. For comparison purposes, the absorbance at 285 nm was rescaled by unit area, so the total area below the curve for both the hydrolysate and its digest was 1 unit. These chromatograms allowed for estimating the molecular weight (MW) distribution of the protein hydrolysates and the digests, reported as a percentage of the total area. The MW distribution was divided into four peptide fractions: A [>10 kDa], B [3–10 kDa], C [1–3 kDa], and D [<1 kDa], as shown in Figure 3.

Overall, the SEC chromatograms showed a shift toward higher elution volumes (i.e., shorter MW) due to the breaking down of initial peptides into lower MW species. The increase in fraction D [<1 kDa] was especially significant for rapeseed meal (Figure 2C), as well as for both protein isolates from pea and potato (Figure 2E,F). All of those digests contained more than 40% wt. of peptides below 1 kDa. These were mainly released from the degradation of medium-size peptides [1–10 kDa] present in the initial hydrolysate. Contrarily, large peptides [>10 kDa] remained mostly unaltered after digestion, which is also reported in the literature [36]. Consequently, their relative proportion in the MW distribution did not change or even increased after digestion at the expense of fractions B [3–10 kDa] and C [1–3 kDa]. Other phenomena, such as the formation of aggregates between peptides and other components by hydrophobic interactions and hydrogen bonds, may contribute to the concentration of large peptide species in the digests, especially for olive seed hydrolysates [21,37].

Most of the potent DPP-IV inhibitory peptides (IC_50_ < 100 mM) identified so far present between 2 and 17 amino acid residues [38]. However, the potential DPP-IV inhibitory effect of a given peptide is mostly explained by its amino acid sequence, which determines its interaction with the DPP-IV active sites [6]. As a common feature, in vitro and in silico studies have shown that the occurrence of hydrophobic amino acids in the peptide sequence may enhance their binding to the hydrophobic pockets present in the S1 subsite of the DPP-IV. More specifically, most of the DPP-IV inhibitory peptides present Pro, Gly, or Leu in proximity or directly attached to the N-terminus [38].

Table 1 presents the amino acid composition of the initial hydrolysates on a molar basis. All the plant protein hydrolysates provided a sufficient amount of essential amino acids (His, Leu, Ile, Lys, Met, Phe, Thr, Try, Val), ranging from 34% (rapeseed) to 45% (potato protein isolate), although it should be noted that tryptophan was not determined by the analysis. Moreover, the combined content of hydrophobic amino acids was maximal for sunflower seed, rapeseed, and potato isolate hydrolysates (45%, 47.5%, and 49% mol, respectively).

The amino acid composition of the digests could be considered to be similar to that of the initial protein hydrolysates since the protein hydrolysates were totally soluble, and thus, all peptides (attacked or not by digestive proteases) would be present in the supernatant obtained after digestion. This was confirmed by the protein content in the insoluble pellets separated after digestion, which was negligible for all the substrates.

### 3.3. DPP-IV Inhibitory Activity of the Hydrolysates and Their Digests

**Figure 4:**
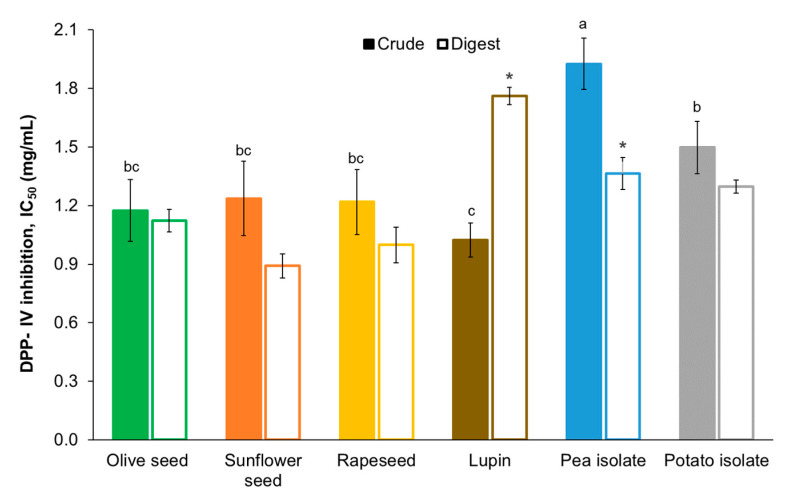
In vitro DPP-IV inhibitory activity (IC50, mg/mL) of the crude protein hydrolysates and their digests. Different superscript letters indicate significant differences (*p* < 0.05) among crude protein hydrolysates. (*) indicates significant differences in IC50 between the crude hydrolysate and its digest.

The antidiabetic potential of hydrolysates was assessed by their potential to inhibit the dipeptidyl peptidase-IV enzyme (DPP-IV). The reported inhibitory activity of food protein hydrolysate usually ranges between 0.03 and 9.17 mg/mL [39]. The inhibitory potential of the hydrolysates produced in this work (Figure 4) was better than other vegetable hydrolysates such as rice (IC_50_ = 2.3 mg/mL) [40] or soy (IC_50_ = 2.39 mg/mL) [41], but lower than hydrolysates from sorghum seeds (IC_50_ = 0.03mg/mL) [10], quinoa (IC_50_ = 0.23 mg/mL) [42], or cowpea bean (IC_50_ = 0.58 mg/mL) [43]. However, the method employed for determining DPP-IV inhibition has an impact on the final IC_50_ value obtained. For example, the concentration of enzyme used for determining the DPP-IV inhibition of quinoa hydrolysates [42] was half that used in the current work. This factor has an impact on the resulting inhibitory activity since it is directly related to the concentration of inhibitory peptides required for reducing enzyme activity.

The inhibitory potential of hydrolysates obtained from agro-industrial by-products was slightly better than the hydrolysates from pea and potato isolates (Figure 4). Generally, peptides with higher molecular weight are related to lower inhibitory activity due to the lower accessibility of large peptides to the active site of DPP-IV [44]. Indeed, the pea hydrolysate, which has the lowest proportion of small-sized peptides below 3 kDa (Figure 3), showed the worst IC_50_. On the contrary, the highest DPP-IV inhibitory activity was obtained for lupin hydrolysate, whose combined content of fractions D [1–3 kDa] and E [<1 kDa] was the highest among the protein sources. DPP-IV inhibitory activity of food-derived peptides is also related to the sequence of amino acids of their chain. Generally, DPP-IV inhibitory peptides have hydrophobic amino acids in their sequence. However, the differences found in the amino acid composition of hydrolysates (Table 1) were not totally related to the observed DPP-IV inhibitory activity (Figure 4). This was attributed to the importance of the specific location of the hydrophobic amino acid residues within the peptide sequence. It has been described that hydrophobic amino acids located in the N-terminal position would enhance the interaction with the active site of DPP-IV, which is required to exert the inhibitory effect [38]. For instance, the location of Pro in the first positions of the N-terminal has been repeatedly reported to positively influence the DPP-IV inhibitory activity of short peptides [6]. Nevertheless, rapeseed hydrolysate, presenting a significantly higher content of Pro (Table 1), did not show a superior inhibition of the DPP-IV enzyme (Figure 4). This finding might be associated with the lower content of short-chain peptides [<1 kDa] in the rapeseed hydrolysate (Figure 3).

When considering the effect of digestion, no significant differences were reported between the DPP-IV inhibitory activity of the original hydrolysates and their digests, except for lupin meal and pea protein isolate. The observed changes in DPP-IV inhibitory capacity after simulated digestion can be related to the modification of the MW distribution (Figure 3). As mentioned before, lower molecular size is related to peptides with higher DPP-IV inhibitory activity and better resistance to gastrointestinal digestion [45]. Pea hydrolysate increased the proportion of low molecular weight peptides [<3 kDa] by around 23% after digestion, which could be related to its improved DPP-IV inhibitory activity. On the contrary, bioactive medium-size peptides [1–3 kDa] present in lupin hydrolysate were extensively degraded during digestion, releasing inactive fragments of lower molecular weight. Rapeseed digest also presented a higher content of short peptides due to the degradation of medium-size bioactive peptides (Figure 3), although the IC_50_ value of the digest was not significantly different compared to the rapeseed hydrolysate (Figure 4). This indicates that, although bioactive peptides could be degraded during digestion, the newly released short-chain species still presented significant levels of DPP-IV inhibitory activity. This might be associated with the high content of Pro in the original protein hydrolysate (Table 1). It is worth noting that although molecular weight distribution can partially describe the inhibitory activity changes observed in digested samples, the amino acid sequence of each peptide is the key parameter determining the DPP-IV inhibitory activity. In this regard, it has been well described that when digestive enzymes hydrolyze active peptides, the resulting fragments could lose partially or totally their inhibitory capacity [46], while in other cases, the resulting fragment could show an improved DPP-IV inhibition [47]. Thus, future studies, including peptidomics, are still required to totally elucidate the effect of digestive proteases on the DPP-IV inhibitory of bioactive peptides present in protein hydrolysates.

## 4. Conclusions

This study reveals that protein hydrolysates exhibiting DPP-IV inhibitory activity could be obtained from meals of agro-industrial by-products such as olive, sunflower, and rapeseed, which do not require any previous isolation process. In addition, the DPP-IV inhibition activity of the hydrolysates obtained from these substrates was not significantly affected during gastrointestinal digestion, which correlates with no significant changes being observed in the molecular weight profile of olive and sunflower hydrolysates after digestion. On the contrary, the bioactivity of lupin and pea hydrolysates was affected during digestion. Although both hydrolysates increased the content of short (<1 kDa) peptides, pea digest showed higher inhibition than pea hydrolysate, while the opposite was observed for lupin. These findings indicate different effects of digestive proteases on DPP-IV inhibitory activity of plant-protein hydrolysates, which is mainly determined by the molecular weight and amino acid sequence of the bioactive peptides present in the undigested hydrolysate. Future studies should include peptidomics to further elucidate the effect of digestion on the composition and content of biopeptides in the hydrolysates. Overall, our results show the potential of using these hydrolysates as bioactive ingredients for the development of functional food exhibiting DPP-IV inhibitory activity.

## Figures and Tables

**Table 1:** Amino acid composition (molar percentage) of the plant protein hydrolysates at DH 20%. Different superscript letters indicate significant differences (*p* < 0.05) among protein substrates for the same amino acid.

Amino Acid	Molar Composition, mol%											
	Olive Seed		Sunflower Seed		Rapeseed		Lupin		Pea Isolate		Potato Isolate	
**Asp**	10.4 ± 0.11	^c^	9.81 ± 0.04	^d^	8.41 ± 0.03	^e^	11.00 ± 0.23	^b^	11.77 ± 0.15	^a^	11.96 ± 0.16	^a^
**Thr**	4.42 ± 0.04	^bc^	4.58 ± 0.01	^b^	4.27 ± 0.09	^c^	4.44 ± 0.08	^bc^	4.22 ± 0.07	^c^	6.22 ± 0.03	^a^
**Ser**	8.07 ± 0.13	^a^	6.45 ± 0.03	^c^	6.53 ± 0.08	^c^	7.41 ± 0.05	^b^	7.07 ± 0.09	^b^	7.19 ± 0.15	^b^
**Glu**	19.36 ± 0.37	^b^	19.97 ± 0.14	^b^	19.46 ± 0.06	^b^	20.76 ± 0.05	^a^	14.99 ± 0.05	^c^	8.94 ± 0.17	^d^
**Pro**	4.99 ± 0.19	^b^	4.92 ± 0.01	^b^	7.83 ± 0.72	^a^	4.93 ± 0.04	^b^	4.89 ± 0.02	^b^	5.53 ± 0.05	^b^
**Gly**	10.03 ± 0.03	^c^	10.98 ± 0.09	^b^	11.84 ± 0.09	^a^	8.36 ± 0.14	^e^	7.28 ± 0.02	^f^	8.91 ± 0.10	^d^
**Ala**	7.72 ± 0.04	^b^	6.92 ± 0.05	^c^	8.61 ± 0.06	^a^	5.79 ± 0.13	^e^	6.51 ± 0.06	^d^	6.81 ± 0.07	^c^
**Cys**	1.85 ± 0.02	^a^	1.12 ± 0.21	^a^	0.98 ± 0.01	^a^	0.85 ± 0.58	^a^	1.05 ± 0.20	^a^	1.40 ± 0.05	^a^
**Val**	6.09 ± 0.32	^a^	5.92 ± 0.07	^a^	3.69 ± 0.06	^b^	4.60 ± 0.35	^b^	5.70 ± 0.10	^a^	6.56 ± 0.28	^a^
**Met**	1.58 ± 0.06	^b^	1.9 ± 0.04	^ab^	1.96 ± 0.08	^ab^	0.50 ± 0.19	^d^	1.06 ± 0.03	^c^	2.18 ± 0.22	^a^
**Ile**	4.11 ± 0.05	^a^	4.15 ± 0.03	^a^	2.52 ± 0.11	^b^	3.98 ± 0.48	^a^	4.57 ± 0.15	^a^	4.73 ± 0.19	^a^
**Leu**	6.39 ± 0.06	^d^	6.29 ± 0.04	^d^	6.30 ± 0.16	^d^	6.80 ± 0.03	^c^	8.11 ± 0.05	^b^	9.26 ± 0.03	^a^
**Tyr**	2.33 ± 0.13	^cd^	1.92 ± 0.1	^d^	2.71 ± 0.18	^bc^	2.48 ± 0.22	^bc^	2.93 ± 0.08	^b^	3.92 ± 0.02	^a^
**Phe**	3.81 ± 0.33	^c^	4.11 ± 0.03	^bc^	4.79 ± 0.11	^a^	3.61 ± 0.04	^c^	4.49 ± 0.02	^ab^	4.84 ± 0.01	^a^
**His**	2.17 ± 0.05	^b^	2.08 ± 0.02	^b^	1.89 ± 0.02	^c^	2.41 ± 0.01	^a^	2.11 ± 0.01	^b^	1.65 ± 0.01	^d^
**Lys**	1.99 ± 0.12	^f^	2.96 ± 0	^e^	3.93 ± 0.03	^d^	4.5 ± 0.08	^c^	6.97 ± 0.02	^a^	6.32 ± 0.02	^b^
**Arg**	4.69 ± 0.08	^c^	5.92 ± 0.04	^d^	4.28 ± 0.07	^e^	7.6 ± 0.17	^a^	6.28 ± 0.01	^b^	3.57 ± 0.02	^f^
**S Hydrophobic AA**	44.72 ± 0.45	^b^	45.19 ± 0.11	^b^	47.54 ± 0.18	^a^	38.57 ± 0.31	^d^	42.61 ± 0.18	^c^	48.82 ± 0.50	^a^
**S Essential AA**	35.25 ± 0.71	^a^	37.91 ± 0.08	^b^	33.63 ± 0.27	^a^	38.44 ± 0.80	^b^	43.51 ± 0.23	^a^	45.33 ± 0.67	^a^

## Data Availability

The original contributions presented in the study are included in the article; further inquiries can be directed to the corresponding author.
